# Effect of facial emotion recognition learning transfers across emotions

**DOI:** 10.3389/fpsyg.2024.1310101

**Published:** 2024-01-19

**Authors:** Taiyong Bi, Wei Luo, Jia Wu, Boyao Shao, Qingli Tan, Hui Kou

**Affiliations:** ^1^Research Center of Humanities and Medicine, Zunyi Medical University, Zunyi, China; ^2^The Institute of Ethnology and Anthropology, Chinese Academy of Social Sciences, Beijing, China

**Keywords:** perceptual learning, facial emotion, visual search, transfer, face recognition

## Abstract

**Introduction:**

Perceptual learning of facial expression is shown specific to the train expression, indicating separate encoding of the emotional contents in different expressions. However, little is known about the specificity of emotional recognition training with the visual search paradigm and the sensitivity of learning to near-threshold stimuli.

**Methods:**

In the present study, we adopted a visual search paradigm to measure the recognition of facial expressions. In Experiment 1 (Exp1), Experiment 2 (Exp2), and Experiment 3 (Exp3), subjects were trained for 8 days to search for a target expression in an array of faces presented for 950 ms, 350 ms, and 50 ms, respectively. In Experiment 4 (Exp4), we trained subjects to search for a target of a triangle, and tested them with the task of facial expression search. Before and after the training, subjects were tested on the trained and untrained facial expressions which were presented for 950 ms, 650 ms, 350 ms, or 50 ms.

**Results:**

The results showed that training led to large improvements in the recognition of facial emotions only if the faces were presented long enough (Exp1: 85.89%; Exp2: 46.05%). Furthermore, the training effect could transfer to the untrained expression. However, when the faces were presented briefly (Exp3), the training effect was small (6.38%). In Exp4, the results indicated that the training effect could not transfer across categories.

**Discussion:**

Our findings revealed cross-emotion transfer for facial expression recognition training in a visual search task. In addition, learning hardly affects the recognition of near-threshold expressions.

## 1 Introduction

Perceptual learning refers to any relatively permanent and consistent change in the perception of objects and their features, following the training of the object and its features (Gibson, 1963). A large number of previous studies have shown that perceptual learning can improve the detection and discrimination of many basic visual features, such as orientation (Hu et al., 2021; Zhang et al., 2023), contrast (Yu et al., 2004; Zhou et al., 2006; Hua et al., 2010; Yu et al., 2016; Roberts and Carrasco, 2022), spatial phase (Berardi and Fiorentini, 1987), stereoacuity (Fendick and Westheimer, 1983; Roberts and Carrasco, 2022), visual acuity (Zhou et al., 2006; Eisen-Enosh et al., 2023), vernier acuity (Fahle and Edelman, 1993), and texture (Karni and Sagi, 1991). Perceptual learning has long been characterized by its specificity to learned features. For example, Ball and Sekuler (1987) found that visual motion learning was direction-specific. Schoups et al. (1995, 2001) found that perceptual learning of orientation was specific to the trained position and the trained orientation. In addition to the learning of elementary features, specificity is also identified in the learning of complex stimuli. For example, object identification learning was specific to the learned set of objects (Furmanski and Engel, 2000); facial viewpoint learning was specific to the learned viewpoint (Bi et al., 2010). However, these kinds of learning usually transfer across retina locations and are little influenced by low-level feature changes. Regarding facial emotions, evidence showed that perceptual learning of facial expression by a facial expression discrimination task was restricted to the trained expression (Du et al., 2016), while Wang et al. (2019) and Wang and Fang (2016) adopting an expression detection task found strong transfer between specific expressions, such as disgust and anger, as well as fear and surprise. Although most studies have revealed strong specificity in various types of learning, it should be noted that they often adopted tasks, like visual discrimination, which require high precision in visual processing. It is worth examining the specificity and transfer of learning in more kinds of training tasks.

Emotional stimuli are usually salient stimuli. Numerous studies have shown that individuals are more likely to direct or allocate their attention to emotional than neutral stimuli in various tasks such as the Stroop task (Richards and Blanchette, 2004; Phaf and Kan, 2007; Dresler et al., 2009; Cisler et al., 2011; Arioli et al., 2021; Amaro-Díaz et al., 2022), the visual search task (Eastwood et al., 2001; Fox, 2002; Frischen et al., 2008; Dodd et al., 2017), and the dot-probe task (Staugaard, 2009; Sutton and Altarriba, 2011; Chapman et al., 2019; Wirth and Wentura, 2020; Fink-Lamotte et al., 2022). Electrophysiological evidence has shown that the EPN (early posterior negativity) and LPP (late positive potential) components can be modulated by the valence in emotional stimuli such as facial expressions (Luo et al., 2010; Smith et al., 2013; Schindler and Bublatzky, 2020; Maffei et al., 2021), affective pictures (Schupp et al., 2003; Huang and Luo, 2006; Solomon et al., 2012; Thom et al., 2014), and emotional words (Kissler et al., 2009; Frühholz et al., 2011). As emotional stimuli are biologically important and salient, they are found to be processed even if they are briefly presented or not consciously perceived. For example, subliminal facial emotions had an impact on the judgments of the subsequent neutral facial expressions (Prochnow et al., 2013). However, some other studies did not reveal the processing of emotional stimuli if they were presented subliminally (Tipura et al., 2019; Baier et al., 2022; Tipura and Pegna, 2022).

The cognitive processing of facial emotions has been shown to be effectively improved by training. Du et al. (2016) trained subjects with a perceptual discrimination task on facial expressions, and they found a significant decrease in the discrimination threshold of the trained expression. Amir et al. (2008, 2009) found that participants with social anxiety and generalized social phobia could be trained to disengage their attention from disgusted faces and to reduce their attentional bias to threatening faces through a modified dot-probe task. Beevers et al. (2015) adopted a similar dot-probe training task, and they found that the training reduced attentional bias to negative faces. Concurrently, training increased resting-state connectivity within the neural circuit consisting of the middle frontal gyrus and dorsal anterior cingulate cortex, indicating that training altered the neural processing of facial emotions. However, little is known about the transfer of the training effect to other stimuli.

In the present study, we adopted the visual search paradigm to train subjects in facial expression recognition. The visual search paradigm has been widely used in training studies. For example, it was found that training to search for happy faces produced a positive bias (Waters et al., 2013), reduced negative bias (De Voogd et al., 2017), and reduced vigilance for threatening stimuli (Dandeneau et al., 2007). To examine the transfer effect of training between different expressions, we trained subjects with one of the two expressions (happy and sad) and tested them on both faces. Similar to Du et al. (2016), happy and sad artificial faces were adopted as experimental stimuli in the present study. However, the present study utilized a visual search task as the training task, while Du et al. (2016) adopted a perceptual discrimination task. The measurement in the present study was the search accuracy, while Du et al. (2016) were concerned with the discrimination threshold. Moreover, to examine how training affects the near-threshold processing of facial emotions, we manipulated the presentation time of the search display.

## 2 Methods

### 2.1 Participants

We conducted a power analysis to determine the sample size through G-power 3.1.9.7. To find a significant (*α* = 0.05) improvement during training, at a level of effect size *d* = 0.7 with a statistical power of 0.95, a sample size of 24 is required. A total of 96 undergraduates were recruited (63 women). Their ages ranged from 18 to 22 (*M* = 20.03, SD = 1.40). Each experiment contained 24 participants. No participant participated in more than one experiment. All participants were right-handed and had normal or corrected-to-normal vision and normal color vision, and none of them had a history of self-reported neurological or psychiatric illness. All procedures performed in this study involving human participants were approved by the Ethical Committee at Zunyi Medical University and were carried out in accordance with the 1964 Declaration of Helsinki and its later amendments. Informed consent was obtained from all participants before participation.

### 2.2 Materials

**Figure 1:**
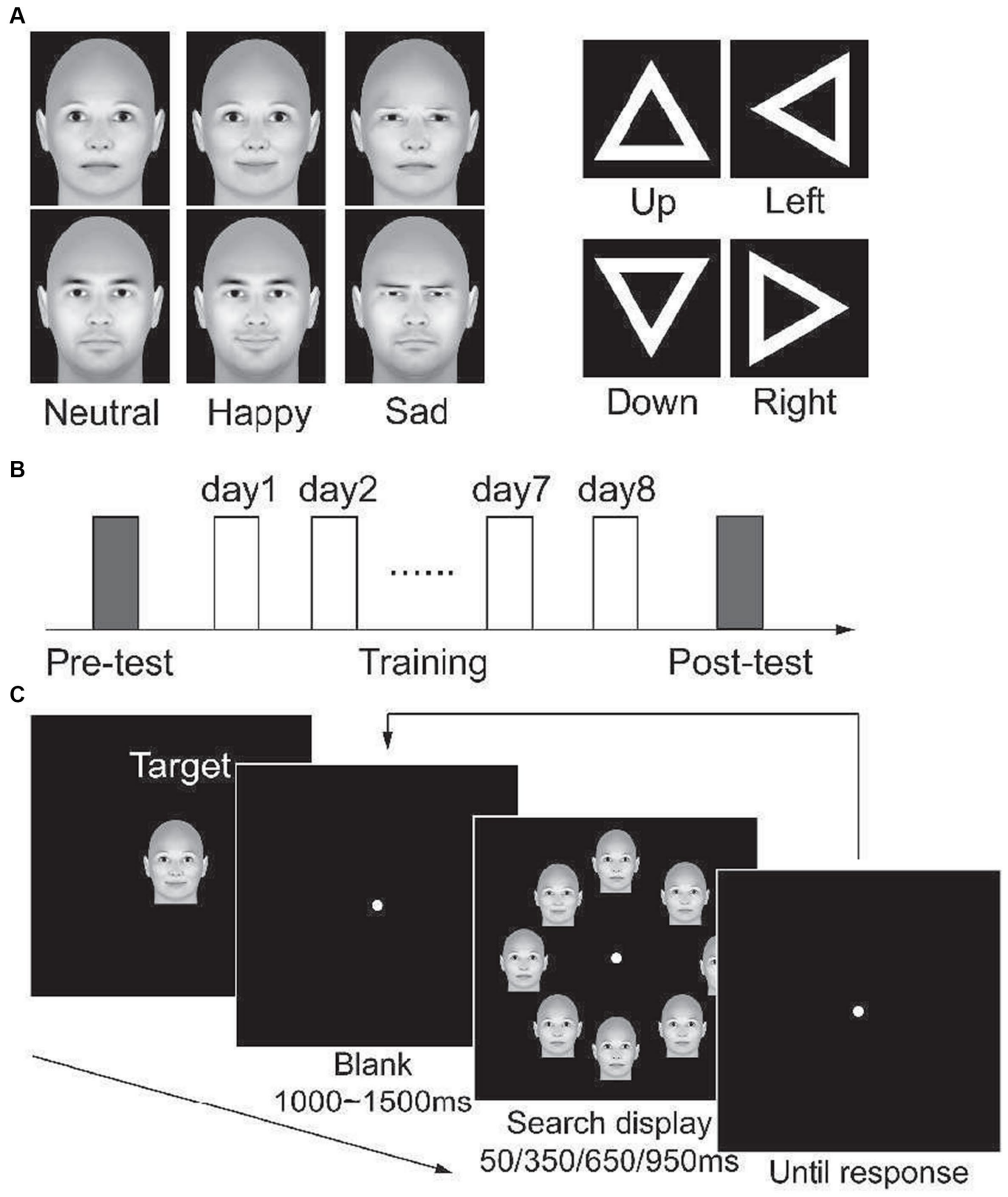
**(A)** demonstrations of experimental stimuli. **(B)** the protocol of all four experiments. On separate days, participants complete the pre-test, 8 days of training, and post-test consecutively. **(C)** a demonstration of the visual search task. At the beginning of each block, the search target of this block is presented. Then, participants were asked to search for the target in a display that included eight stimuli in each trial.

Three-dimensional (3D) face models were generated by FaceGen Modeler 3.1 (http://www.facegen.com). No hair was rendered. A male face model and a female face model were generated by setting the gender slider to male and female, respectively. Other parameters were left in the default setting for neutral faces. Faces with happy or sad expressions were created with the setting Expression: SmileClosed or Expression: Sad to 1, respectively. Face models are illustrated in Figure 1A. Visual stimuli were generated by projecting a 3D stimulus model with the front view onto the monitor plane. The stimuli extended 5.6 × 4.5° of visual angle. They were presented on a 19-inch LCD monitor, with a spatial resolution of 1,024 × 768 and a refresh rate of 60 Hz (Zhang et al., 2018). Subjects viewed the stimuli from a distance of 60 cm. Their head position was stabilized using a chin rest and a headrest. Throughout the experiments, subjects were asked to fixate on a small green dot presented at the center of the monitor.

Other than the face stimuli, we created triangles for Exp 4. As Figure 1A illustrates, the stimuli are white hollow equilateral triangles. The outer length of each side was 3.7°, and the width of the side was 0.56°. We created four triangles that oriented left, right, up, and down, respectively.

### 2.3 Procedure

The present study consisted of four experiments. In the first three experiments, we trained participants to search for an emotional face (target) among seven neutral faces (distractor), with the stimuli displayed for 950 ms, 350 ms, and 50 ms in Experiment 1 (Exp1), Experiment 2 (Exp2), and Experiment 3 (Exp3), respectively. In Experiment 4 (Exp4), we trained participants to search a target triangle within 350 ms. All the experiments included three phases (Figure 1B), i.e., a pre-training test phase (pre-test), a training phase during 8 days, and a post-training test phase (post-test).

#### 2.3.1 Experiment 1

**Figure 2:**
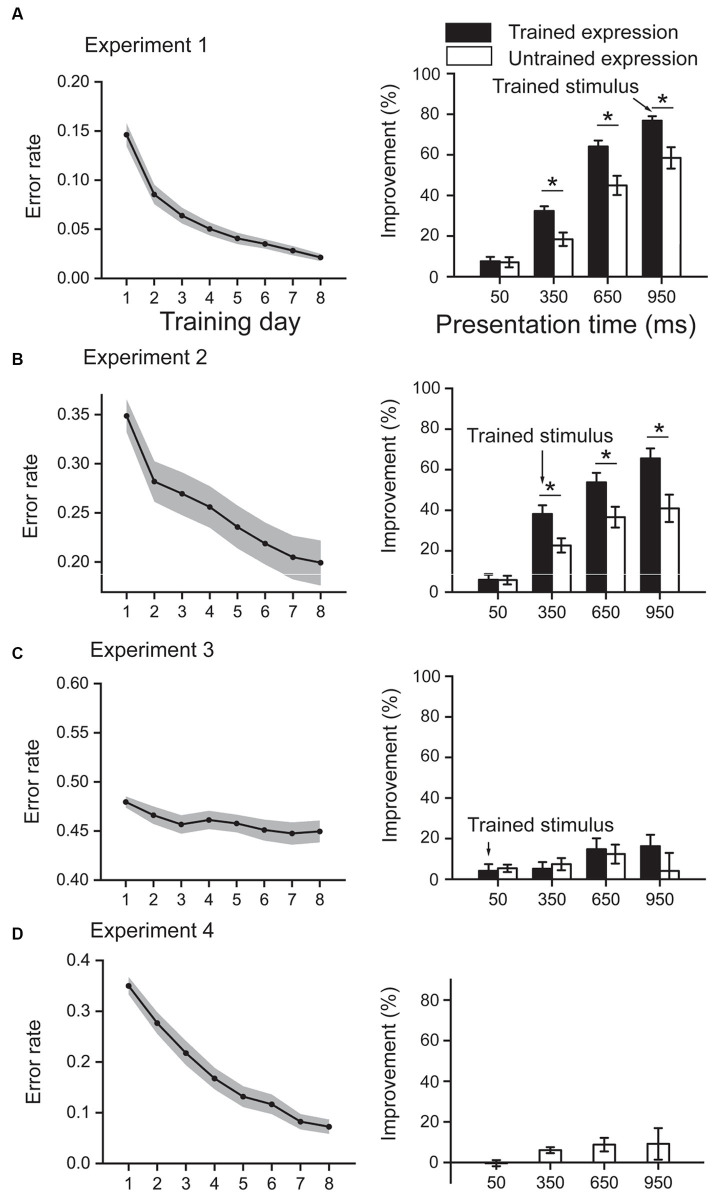
Training effect results. **(A)** results from Experiment 1, in which the training stimuli are emotional faces, were presented for 950 ms. **(B)** results from Experiment 2, in which the training stimuli are emotional faces, were presented for 350 ms. **(C)** results from Experiment 3, in which the training stimuli are emotional faces, were presented for 50 ms. **(D)** results from Experiment 4, in which the training stimuli are triangles, were presented for 350 ms. In each experiment, the left panel shows the error rate during the training phase; the right panel shows the improvement between post- and pre-tests. Shaded areas in the left panels and error bars in the right panels denote one standard error of the mean. *p < 0.05, Bonferroni corrected.

In the pre-test phase, participants were tested with a visual search task. As illustrated in Figure 1C, each trial began with a fixation of 1,000 ms ~ 1,500 ms. A search display was then presented for a period of 50 ms, 350 ms, 650 ms, or 950 ms. The search display consisted of eight faces. Four of the faces were placed beyond, under, left to, and right to the fixation, while the other four faces were placed in the four quadrants. The distance between the center of each face and fixation was 9°, and the distance between the centers of two neighboring faces was also 9°. Participants were asked to press keys to indicate whether there was a target among distractors as soon and accurately as possible. Each block contained 48 trials, with 12 trials for each presentation time. Half of the trials contained a target, while the target was absent in the other trials. In each block, the search target was fixed and presented before the task started. Each participant completed eight blocks for each search target and completed a total of 32 blocks in the pre-test phase. Male and female faces were presented in separate blocks. The order of blocks was counterbalanced (Figure 2).

**Figure 3:**
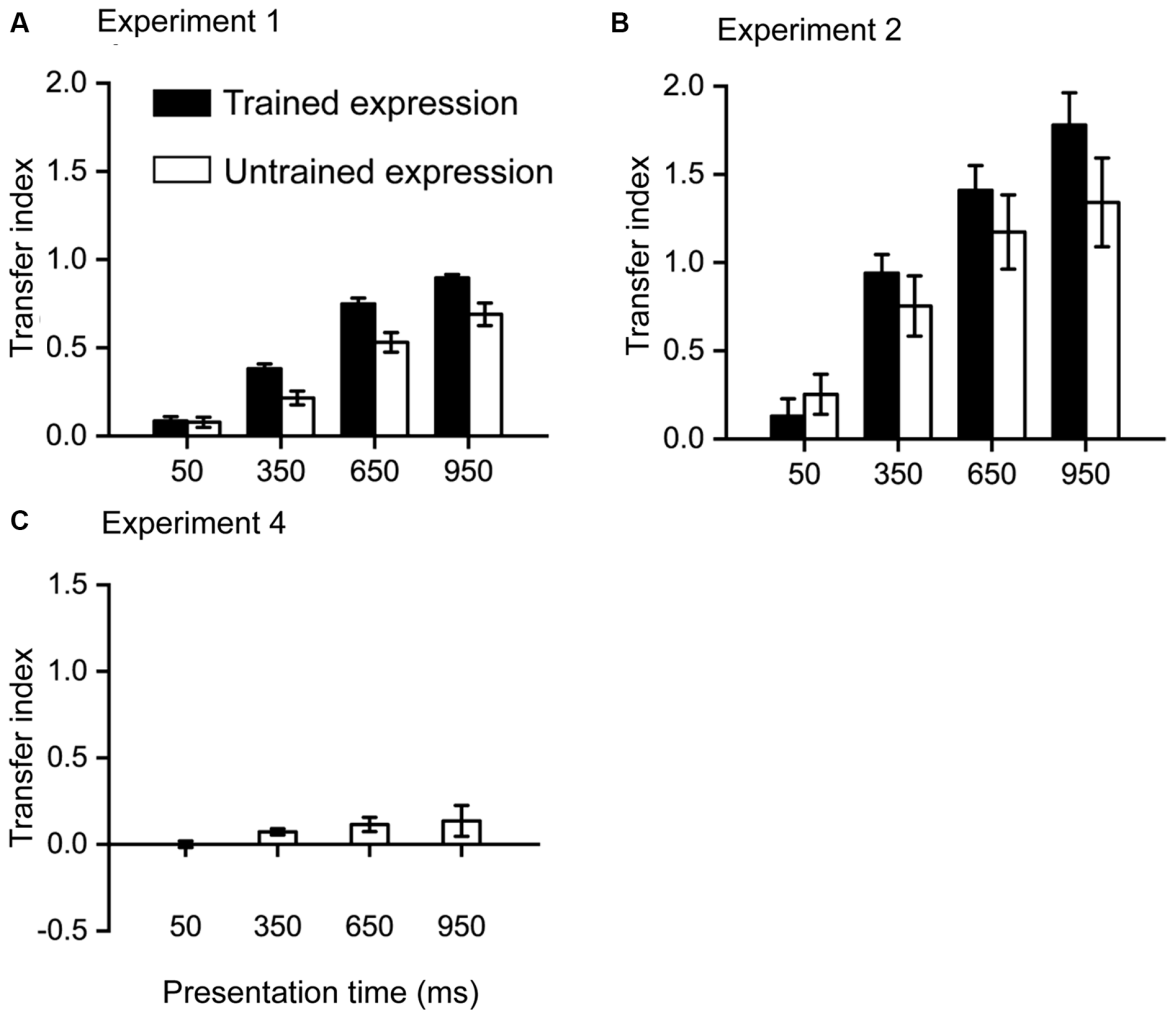
Transfer index results. **(A)** results from Experiment 1, in which the training stimuli are emotional faces, were presented for 950 ms. **(B)** results from Experiment 2, in which the training stimuli are emotional faces, were presented for 350 ms. **(C)** Results from Experiment 4, in which the training stimuli are triangles, were presented for 350 ms. Error bars denote one standard error of the mean.

In the training phase, each participant was assigned to search a specific search target. Participants completed 20 blocks on each training day. Each block contained 48 trials. The procedure of each trial was similar to the task in the test phase. However, the presentation time for each display was fixed at 950 ms. Furthermore, a beep feedback was presented when a wrong response was made (Figure 3).

On the next day after the training, participants completed post-test tasks, which were the same as the pre-test tasks.

#### 2.3.2 Experiment 2 and experiment 3

The tasks in the test phases were the same as in Exp1. The only difference was the presentation time of the search display in the training phase. In Exp2, the presentation time was 350 ms. In Exp3, the presentation time was 50 ms.

#### 2.3.3 Experiment 4

The tasks in the test phases were the same as in Exp1. In the training phase, a shape search task was adopted. Each participant was assigned to search for one of the four triangles. The search display consists of eight triangles with the same arrangement as the face display. In trials with the target presented, one of the eight triangles was the target, while other triangles were randomly selected from the non-target triangles. The display was presented for 350 ms. Participants were also asked to indicate whether the target was presented. A beep feedback was provided whenever a mistake was made.

### 2.4 Statistical analysis

First, we analyzed the error rate (Err) and reaction time (RT). In the training phase, the error rate and RT of each day were calculated based on the data of the 20 blocks. In the test phases, trials were divided into two conditions: trained expression and untrained expression. For example, for the participants who were trained with female sad faces or male sad faces, sad faces were the trained condition, while happy faces were the untrained condition. Error rate and RT were calculated for each condition and each presentation time. Further statistical analyses were based on the improvements in the performance (Bi et al., 2010). The improvements were calculated as the difference between the performance in the two test phases compared to the baseline performance in the pre-test. For example, for the improvement in the trained expression condition, Err_test_TE_imp_ = (Err_test_TE_pre_ - Err_test_TE_post_) / Err_test_TE_pre_ × 100%, where Err_test_TE_imp_ is the improvement of the error rate in the trained expression condition between pre- and post-tests. A positive index indicates that the error rate was lower in the post-test than in the pre-test. Similarly, we can also calculate the improvement of the error rate in the untrained expression condition as Err_test_UE_imp_. Afterward, 2 (Expression: trained and untrained) × 4 (presentation time: 50 ms, 350 ms, 650 ms, and 950 ms) repeated measures ANOVAs were conducted on the improvement of error rate and the improvement of RT. In the same way, the improvement in the training phase could be defined as the difference between the first day and the last day of training, compared to the baseline performance on the first day of training. For example, Err_training_imp_ = (Err_day1_ - Err_day8_) / Err_day1_ × 100%, where day1 is the first day of training, and day8 is the last day of training.

Second, to directly examine the transfer effect from the training phase to the test phase, we defined a transfer index (Bi et al., 2010). This index was calculated as the ratio of the improvement in the test phases over the improvement in the training phase. For example, the transfer index for the trained expression condition is calculated as TI_TE = Err_test_TE_imp_ / Err_training_imp_. Similarly, the transfer index for the untrained expression condition is calculated as TI_UE = Err_test_UE_imp_ / Err_training_imp_. The TI indicated the proportion of the training effect that transferred to a specific condition.

The following three points should be noted. First, as we found quite a small training effect in Exp3, the TI could not be calculated because Err_training_imp_ was too small. Second, because we did not train participants with face stimuli in Exp4, we could not divide faces in the test phases into trained and untrained expression conditions. Therefore, we pooled the results across different face stimuli together, resulting in only one factor, the presentation time, in Exp4. Third, as the main training effects were only evident in error rate results, TIs were only calculated based on the error rate. No TI for RT results was calculated.

## 3 Results

### 3.1 Experiment 1

In this experiment, participants were trained with emotional faces presented for 950 ms (Figure 2A). During the 8-day training, the error rate decreased from 0.15 to 0.02 [*t* (23) = 12.56, *p* < 0.001], resulting in an improvement of 85.89%. A 2 (Expression: trained and untrained) × 4 (Presentation time: 50 ms, 350 ms, 650 ms, and 950 ms) repeated-measures ANOVA on the improvements of error rate in test phases showed that the interaction effect was significant [*F* (3, 69) = 6.36, *p* = 0.001, η_p_^2^ = 0.217]. Simple effect analysis showed that the improvements were higher for the trained expression than untrained expression at the presentation time of 350 ms, 650 ms, and 950 ms (all *p* < 0.05, Bonferroni corrected), while the difference was non-significant at 50 ms (*p* = 0.871). In addition, the main effect of expression and presentation time was both significant (both *F* > 15, *p* < 0.001, η_p_^2^ > 0.3).

Regarding RT, an ANOVA on the improvements showed a non-significant interaction effect [*F* (3, 69) = 2.13, *p* = 0.104, η_p_^2^ = 0.085]. The main effects of expression and presentation time were both non-significant (both *F* < 0.3, *p* > 0.6, η_p_^2^ < 0.01).

### 3.2 Experiment 2

In this experiment, participants were trained with emotional faces presented for 350 ms (Figure 2B). During the 8-day training, the error rate decreased from 0.35 to 0.20 [*t* (23) = 9.74, *p* < 0.001], resulting in an improvement of 46.05%. A 2 (Expression: trained and untrained) × 4 (Presentation time: 50 ms, 350 ms, 650 ms, and 950 ms) repeated-measures ANOVA on the improvements of error rate in test phases showed that the interaction effect was significant [*F* (3, 69) = 7.53, *p* < 0.001, η_p_^2^ = 0.247]. Simple effect analysis showed that the improvements were higher for the trained expression than untrained expression at the presentation time of 350 ms, 650 ms, and 950 ms (all *p* < 0.05, Bonferroni corrected), while the difference was non-significant at 50 ms (*p* = 0.949). In addition, the main effect of expression and presentation time was both significant (both *F* > 12, *p* < 0.01, η_p_^2^ > 0.3).

Regarding RT, an ANOVA on the improvements showed a non-significant interaction effect [*F* (3, 69) = 2.39, *p* = 0.076, η_p_^2^ = 0.094]. The main effects of expression and presentation time were both non-significant (both *F* < 0.6, p > 0.6, η_p_^2^ < 0.03).

### 3.3 Experiment 3

In this experiment, participants were trained with emotional faces presented for 50 ms (Figure 2C). During the 8-day training, the error rate slightly decreased from 0.48 to 0.45 [*t* (23) = 3.54, *p* = 0.002], resulting in only an improvement of 6.38%. A 2 (Expression: trained and untrained) × 4 (Presentation time: 50 ms, 350 ms, 650 ms, and 950 ms) repeated-measures ANOVA on the improvements of error rate in test phases showed that the interaction effect was non-significant [*F* (3, 69) = 2.12, *p* = 0.105, η_p_^2^ = 0.085]. In addition, the main effect of expression and presentation time was non-significant (both *F* < 1.8, *p* > 0.1, η_p_^2^ < 0.07).

Regarding RT, an ANOVA on the improvements showed a non-significant interaction effect [*F* (3, 69) = 0.40, *p* = 0.752, η_p_^2^ = 0.017]. The main effects of expression and presentation time were both non-significant (both *F* < 0.8, *p* > 0.3, η_p_^2^ < 0.04).

### 3.4 Experiment 4

In this experiment, participants were trained with triangles presented for 350 ms (Figure 2D). During the 8-day training, the error rate decreased from 0.35 to 0.07 [*t* (23) = 21.89, *p* < 0.001], resulting in an improvement of 81.62%. A single factor (presentation time) repeated-measures ANOVA on the improvements in test phases showed that the main effect was non-significant [*F* (3,69) = 1.52, *p* = 0.217, η_p_^2^ = 0.062].

Regarding RT, an ANOVA on the improvements showed a non-significant main effect [*F* (3,69) = 0.75, *p* = 0.529, η_p_^2^ = 0.031].

### 3.5 Transfer index

The transfer index quantifies the learning effect in each condition as the proportion of the improvement during the training phase. A larger index indicates more benefits in a condition from training. As Exp3 found only a small training effect, it is unnecessary to calculate TI for this experiment.

In Exp1 (Figure 3A), the highest TI was the trained expression at 950 ms, indicating that 89.54% of the training effect was transferred to this condition. All other TIs were significantly lower than this TI [all *t* (23) > 3.3, *p* < 0.05, Bonferroni corrected]. The lowest TI was the untrained expression at 50 ms, indicating that only 7.80% of the training effect was transferred to this condition. The TI for the trained expression at 50 ms (8.45%) was not significantly higher than this TI [*t* (23) = 0.175, *p* = 0.863], while all other TIs were significantly higher than this TI (all *t* > 3.3, *p* < 0.05, Bonferroni corrected).

In Exp2 (Figure 3B), the highest TI was the trained expression at 950 ms, indicating that 177.91% of the training effect was transferred to this condition. All other TIs were significantly lower than this TI [trained expression at 950 ms: *t* (23) = 2.83, *p* = 0.010, uncorrected; other *t* (23) > 3.7, *p* < 0.05, Bonferroni corrected]. The lowest TI was the trained expression at 50 ms, indicating that only 12.95% of the training effect was transferred to this condition. The TI for the untrained expression at 50 ms (25.26%) was not significantly higher than this TI [*t* (23) = 1.62, *p* = 0.119], while all other TIs were significantly higher than this TI (all *t* > 3.7, *p* < 0.05, Bonferroni corrected).

In Exp4 (Figure 3C), the highest TI was at 950 ms, indicating that 13.60% of the training effect was transferred to this condition. All other TIs were non-significantly different from this TI [all *t* (23) < 1.8, *p* > 0.1].

## 4 Discussion

In the present study, we found that when the stimuli were presented long enough, training could significantly reduce the errors in searching for an emotional face, reflecting an enhancement in the recognition of facial emotions. Furthermore, such training effects were modulated by the presentation time of the search display. Generally, the improvements increased with the presentation time, which means that the most prominent improvement may not always be the trained condition. A crucial finding of the present study is that the training effect could transfer to the untrained expression, indicating that learning may happen at a more general level of emotional information processing. However, when the stimuli were presented briefly, small learning effects were found, suggesting that learning requires stable processing of the emotional stimuli. Finally, training effects could only transfer within the face category. The category-specific training effect ruled out the explanation that a general ability of attention was enhanced by the training.

We found that training can improve the ability to recognize facial expressions, consistent with previous studies (Du et al., 2016; Peters et al., 2017; Penton-Voak et al., 2018; Hiemstra et al., 2019; Penton-Voak et al., 2020; Wells et al., 2021; Haller et al., 2022). More importantly, we found that the effects of training were not restricted to the trained expression. This finding was inconsistent with a previous study adopting a facial expression discrimination task (Du et al., 2016), in which the training effect showed strong specificity to the trained expression. However, some other studies adopting an expression detection task revealed strong transfer between specific expressions, such as disgust and anger, as well as fear and surprise (Wang and Fang, 2016; Wang et al., 2019). Interestingly, they found an asymmetric transfer effect between happy and sad faces, i.e., the training effect could transfer from sad faces to happy faces, but the reverse was not true. An evident difference among these studies was the task of training. Du et al. (2016) examined the discrimination performance, while Wang et al. (2019) and Wang and Fang (2016) examined the detection of expression from external noise. In the present study, the visual search task was adopted. Taken together, the higher generality of the training effect in our study suggests that visual search training may improve higher-level cognitive functions to facial expression rather than lower-level expression processing.

Although the topic of the present study is similar to the previous study performed by Du et al. (2016), several substantial differences need to be noted. First, the population of the present study was different from the previous one. Second, the main task of the present study is visual search, while the previous study adopted a perceptual discrimination task. The core cognitive component may be different between the two tasks. The visual search performance depends on the sensitivity to the salience of the target stimulus, while the discrimination threshold depends on the sensitivity to the variations of the stimulus. Third, the main finding of the present study revealed strong transfers across different facial expressions, indicating that the training on the sensitivity of stimulus salience may not depend on the trained stimuli. However, Du et al. (2016) found a strong specificity of the training to the trained expression, indicating that the training may change specific stimulus representation. Finally, we also examined the training effect on briefly presented expressions, while Du et al. (2016) only examined supraliminal expressions. In summary, our study investigated the training effect on different cognitive processing from the previous study and revealed a more transferable training effect. It should be noted that the stimuli used in the present study and Du et al.’s study were identical. Therefore, different mechanisms between different tasks may contribute to the two different training effects.

The visual search paradigm has been extensively used to train the recognition of facial expressions (Dandeneau et al., 2007; Kruijt et al., 2013; Waters et al., 2013; De Voogd et al., 2017). However, previous studies mainly focused on cognitive modifications among specific populations. For example, De Voogd et al. (2017) adopted an online visual search task to train adolescents with heightened anxiety and depressive symptoms to find a happy face among negative faces (angry, fearful, and sad), and they found that the visual search training reduced the negative bias. In the present study, we are more concerned about the general mechanisms underlying the visual search training and found general enhancements in facial emotion recognition. Our findings showed the transfer or generalization of recognition training. However, we did not test the transfer between tasks, which requires further investigation.

An overall improvement in recognition was excluded by Exp4, so the training might improve the recognition only for facial expressions. There might be two major neural systems involved in emotional processing: a subcortical, ‘bottom-up’ amygdala-based system and a cortical, ‘top-down’ cognitive control system (March, 2010). Vuilleumier (2005) proposed that enhanced perceptual processing of emotional stimuli might result from direct ‘feedback’ signals imposed by the amygdala on cortical pathways, which potentially supplement or compete with other top-down control on perception imposed by attentional systems in the frontal and parietal cortex. Improved recognition of facial expressions may be associated with changes in both bottom-up and top-down pathways. However, it is still unclear whether the visual search training alters these neural responses.

Another interesting finding of the present study is that the recognition of briefly presented facial expressions was little affected by training, suggesting that learning requires stable processing of emotional content in the stimulus. When the search display was presented for only 50 ms, subjects showed near-threshold performance (accuracies ranged from 51.41 to 53.04% in the four experiments) in recognizing an emotional face, indicating insufficient processing of facial emotions. Even after 8 days of training, the recognition (55% accuracy) was still far below the threshold performance. Although subliminal processing of emotional stimuli was revealed in some studies (MacLeod and Hagan, 1992; Mogg et al., 1993; Van Den Hout et al., 1995; Bornemann et al., 2012), there was also contradictory evidence (Tipura et al., 2019; Baier et al., 2022; Tipura and Pegna, 2022). Furthermore, it is unclear whether the subliminal processing of facial emotions could be affected by training. Our findings suggest that the training has little influence on the processing of near-threshold facial expressions.

## 5 Limitations

First, the present study only examined the training effects of happy and sad faces. Further studies should be conducted to examine the training effects of more facial emotions and the transfer among them. For example, threatening faces may induce more evident processing. A behavioral study has revealed that subliminal angry faces made participants more likely to voluntarily withhold the action, whereas fearful and happy faces had no effects (Parkinson et al., 2017). Therefore, it is worth trying to train subjects with threatening stimuli, such as angry faces, fearful faces, and threatening animals. Second, the neural mechanism of the emotional training effect is still unclear. Previous neuroimaging studies usually adopted the dot-probe task and examined the neural mechanisms of the training effect on attentional bias (Eldar and Bar-Haim, 2010; Osinsky et al., 2014; Beevers et al., 2015; Britton et al., 2015). The neural mechanism of visual search training may be different and requires further investigation.

## Data availability statement

The raw data supporting the conclusions of this article will be made available by the authors, without undue reservation.

## Ethics statement

The studies involving humans were approved by Ethical Committee at Zunyi Medical University. The studies were conducted in accordance with the local legislation and institutional requirements. The participants provided their written informed consent to participate in this study.

## Author contributions

TB: Funding acquisition, Investigation, Methodology, Project administration, Resources, Writing – original draft. WL: Conceptualization, Methodology, Supervision, Validation, Writing – original draft. JW: Data curation, Investigation, Project administration, Writing – original draft. BS: Formal analysis, Investigation, Software, Writing – original draft. QT: Data curation, Investigation, Writing – original draft. HK: Conceptualization, Funding acquisition, Resources, Supervision, Validation, Writing – review & editing.
