# Supplementary material for: Effect of facial emotion recognition learning transfers across emotions
Source: Front Psychol. 2024 Jan 19;15:1310101. doi: 10.3389/fpsyg.2024.1310101 (PMC10834736; doi:10.3389/fpsyg.2024.1310101)
Supplement: Supplementary file 1 [file Data_Sheet_1.docx]

Supplementary results

Table S1 Error rate and RT results in Exp1: M(SD)

|  |  | Trained expression | | Untrained expression | |
| --- | --- | --- | --- | --- | --- |
|  | Presentation time | Pre-test | Post-test | Pre-test | Post-test |
| Error rate | 50ms | 0.48(0.03) | 0.44(0.05) | 0.47(0.04) | 0.44(0.04) |
|  | 350ms | 0.40(0.07) | 0.27(0.056) | 0.40(0.07) | 0.32(0.05) |
|  | 650ms | 0.35(0.10) | 0.12(0.064) | 0.32(0.11) | 0.18(0.08) |
|  | 950ms | 0.29(0.12) | 0.07(0.05) | 0.28(0.14) | 0.11(0.07) |
| RT (s) | 50ms | 0.62(0.18) | 0.76(0.17) | 0.62(0.16) | 0.74(0.13) |
|  | 350ms | 0.77(0.21) | 0.89(0.15) | 0.77(0.21) | 0.89(0.10) |
|  | 650ms | 0.90(0.24) | 1.00(0.15) | 0.90(0.25) | 1.02(0.09) |
|  | 950ms | 1.00(0.29) | 1.07(0.13) | 1.00(0.30) | 1.13(0.12) |

Results from Exp1:

We performed a 2 (Session: Pre-test, Post-test) * 2 (Emotion: Trained, Untrained) * 4 (Presentation time: 50ms, 350ms, 650ms, 950ms) repeated measures ANOVA on the error rate data. Results showed a significant interaction among the three factors (F(3, 69)=4.38, p=0.007). Then, two 2 (Emotion: Trained, Untrained) * 4 (Presentation time: 50ms, 350ms, 650ms, 950ms) repeated measures ANOVAs were performed separately for the two test sessions. At pre-test, the interaction between emotion and presentation time was nonsignificant (F(3, 69)=0.81, p=0.491). The main effect of presentation time was significant (F(3, 69)=69.18, p<0.001), while that of emotion was nonsignificant (F(1, 23)=1.06, p=0.314). At post-test, the interaction between emotion and presentation time was significant (F(3, 69)=6.852, p<0.001). Simple effect analysis revealed that the error rates were higher for the trained expression than the untrained expression at the presentation time of 350ms, 650ms and 950ms (all p<0.05, uncorrected), while the difference was nonsignificant at the presentation time of 50ms (p>0.05).

Table S2 Error rate and RT results in Exp2: M(SD)

|  |  | Trained expression | | Untrained expression | |
| --- | --- | --- | --- | --- | --- |
|  | Presentation time | Pre-test | Post-test | Pre-test | Post-test |
| Error rate | 50ms | 0.47(0.04) | 0.44(0.06) | 0.49(0.04) | 0.45(0.04) |
|  | 350ms | 0.41(0.08) | 0.25(0.09) | 0.42(0.07) | 0.32(0.07) |
|  | 650ms | 0.35(0.14) | 0.16(0.09) | 0.37(0.10) | 0.24(0.11) |
|  | 950ms | 0.31(0.16) | 0.11(0.08) | 0.32(0.15) | 0.18(0.11) |
| RT (s) | 50ms | 0.67(0.12) | 0.69(0.17) | 0.67(0.14) | 0.64(0.13) |
|  | 350ms | 0.85(0.17) | 0.80(0.18) | 0.82(0.15) | 0.78(0.10) |
|  | 650ms | 0.97(0.19) | 0.94(0.22) | 0.95(0.19) | 0.93(0.15) |
|  | 950ms | 1.09(0.24) | 1.04(0.22) | 1.08(0.25) | 1.03(0.19) |

Results from Exp2:

We performed a 2 (Session: Pre-test, Post-test) * 2 (Emotion: Trained, Untrained) * 4 (Presentation time: 50ms, 350ms, 650ms, 950ms) repeated measures ANOVA on the error rate data. Results showed a significant interaction among the three factors (F(3, 69)=3.85, p=0.013). Then, two 2 (Emotion: Trained, Untrained) * 4 (Presentation time: 50ms, 350ms, 650ms, 950ms) repeated measures ANOVAs were performed separately for the two test sessions. At pre-test, the interaction between emotion and presentation time was nonsignificant (F(3, 69)=0.10, p=0.957). The main effect of presentation time was significant (F(3, 69)=32.60, p<0.001), while that of emotion was nonsignificant (F(1, 23)=2.84, p=0.105). At post-test, the interaction between emotion and presentation time was significant (F(3, 69)=7.49, p<0.001). Simple effect analysis revealed that the error rates were higher for the trained expression than the untrained expression at the presentation time of 350ms, 650ms and 950ms (all p<0.05, uncorrected), while the difference was nonsignificant at the presentation time of 50ms (p>0.05).

Table S3 Error rate and RT results in Exp3: M(SD)

|  |  | Trained expression | | Untrained expression | |
| --- | --- | --- | --- | --- | --- |
|  | Presentation time | Pre-test | Post-test | Pre-test | Post-test |
| Error rate | 50ms | 0.48(0.04) | 0.46(0.07) | 0.48(0.04) | 0.46(0.03) |
|  | 350ms | 0.39(0.06) | 0.37(0.08) | 0.41(0.08) | 0.37(0.07) |
|  | 650ms | 0.35(0.10) | 0.30(0.12) | 0.35(0.11) | 0.30(0.10) |
|  | 950ms | 0.30(0.13) | 0.24(0.13) | 0.31(0.15) | 0.26(0.11) |
| RT (s) | 50ms | 0.75(0.15) | 0.68(0.22) | 0.79(0.21) | 0.67(0.21) |
|  | 350ms | 0.90(0.18) | 0.82(0.18) | 0.92(0.19) | 0.82(0.21) |
|  | 650ms | 1.06(0.21) | 0.96(0.22) | 1.07(0.23) | 0.93(0.23) |
|  | 950ms | 1.16(0.27) | 1.04(0.25) | 1.20(0.31) | 1.04(0.27) |

Results from Exp3:

We performed a 2 (Session: Pre-test, Post-test) * 2 (Emotion: Trained, Untrained) * 4 (Presentation time: 50ms, 350ms, 650ms, 950ms) repeated measures ANOVA on the error rate data. Results showed a nonsignificant interaction among the three factors (F(3, 69)=0.22, p=0.881). The interactions between any two factors were nonsignificant (all F<2.2, p>0.09). The main effect of presentation time was significant (F(3, 69)=92.60, p<0.001), the main effect of session was significant (F(1, 23)=10.51, p=0.004), while that of emotion was nonsignificant (F(1, 23)=0.51, p=0.484).

Table S4 Error rate and RT results in Exp4: M(SD)

|  | Presentation time | Pre-test | Post-test |
| --- | --- | --- | --- |
| Error rate | 50ms | 0.47(0.03) | 0.47(0.03) |
|  | 350ms | 0.40(0.06) | 0.38(0.07) |
|  | 650ms | 0.34(0.11) | 0.31(0.12) |
|  | 950ms | 0.29(0.13) | 0.26(0.14) |
| RT (s) | 50ms | 0.63(0.15) | 0.60(0.17) |
|  | 350ms | 0.78(0.22) | 0.76(0.22) |
|  | 650ms | 0.91(0.26) | 0.88(0.28) |
|  | 950ms | 1.00(0.32) | 0.96(0.30) |

Results from Exp4:

We performed a 2 (Session: Pre-test, Post-test) * 4 (Presentation time: 50ms, 350ms, 650ms, 950ms) repeated measures ANOVA on the error rate data. The interaction between emotion and presentation time was significant (F(3, 69)=3.28, p=0.026). Simple effect analysis revealed that the error rates were higher for the pre-test session than the post-test session at the presentation time of 350ms and 650ms (both p<0.05, uncorrected), while the differences were nonsignificant at the presentation time of 50ms and 950ms (both p>0.05).
